# Nationwide epidemiological survey of acute pancreatitis in Japan, 2021: the impact of the COVID-19 pandemic and revised clinical guidelines

**DOI:** 10.1007/s00535-025-02284-2

**Published:** 2025-07-31

**Authors:** Yuichi Tanaka, Atsushi Masamune, Ryotaro Matsumoto, Tetsuya Takikawa, Yu Tanaka, Shin Hamada, Shin Miura, Kiyoshi Kume, Yoshifumi Takeyama, Kazuhiro Kikuta, Tohoku University Graduate School of Medicine, Toshiaki Abe, Takahiro Abe, Makoto Abue, Takumi Akiyama, Toshihiko Arizumi, Shohei Asada, Shunjiro Azuma, Kazuro Chiba, Takahiro Dohmen, Hiroyoshi Doi, Takaaki Eguchi, Hiroyuki Endo, Nao Fujimori, Yuko Fujimoto, Junichi Fujiwara, Akihisa Fukuda, Naoya Fukuda, Yoshihisa Fukuda, Masataka Fukue, Hiroyuki Funayama, Toshifumi Gushima, Shin-Ichiro Hagiwara, Hideaki Hamano, Yasushi Hamaya, Kiyotaka Hashizume, Kenji Hattori, Nobuhiro Hattori, Aoi Hayasaki, Hidehiro Hayashi, Kazuki Hayashi, Kazunao Hayashi, Koki Hayashi, Sumio Hirose, Masashi Hirota, Morihisa Hirota, Takashi Hisamatsu, Yasuki Hori, Tatsuya Hoshi, Naoko Hyodo, Kazunari Ibusuki, Akihito Ida, Takao Iemoto, Takamichi Igarashi, Takeshi Iida, Sadaharu Iio, Yuta Iizawa, Tsukasa Ikeura, Tsunao Imamura, Tomoko Inagaki, Osamu Inatomi, Hiroyuki Inoue, Kentaro Inoue, Tadahisa Inoue, Kuniyasu Irie, Shoichi Irie, Masaharu Ishida, Kazunori Ishige, Akio Ishii, Jun Ishii, Hajime Isomoto, Shuichi Ito, Takashi Ito, Tetsuhide Ito, Eisuke Iwasaki, Junji Iwasaki, Takuji Iwashita, Keisuke Iwata, Tomoyuki Iwata, Toshinobu Izumi, Naruomi Jinno, Takuo Kado, Seiji Kaino, Tetsuro Kajiwara, Toyoma Kaku, Takahiro Kamiga, Akira Kanamori, Keiko Kaneko, Katsuhiro Kanemaru, Atsushi Kanno, Satoshi Kasugai, Fumiya Kataoka, Shin Kato, Junji Kawaguchi, Shinya Kawaguchi, Kenji Kawaguti, Ryuichi Kawahara, Yoshinari Kawahara, Tomoaki Kawai, Yuki Kawaji, Hiroshi Kawakami, Toru Kawamoto, Osamu Kido, Yui Kishimoto, Hideki Kitada, Takanori Kitaguchi, Katsuya Kitamura, Rena Kitano, Kasenn Kobashigawa, Masanori Kobayashi, Ikuhiro Kobori, Yuzo Kodama, Shiro Kohi, Kazuhiko Koike, Mitsuhito Koizumi, Takamitsu Komaki, Shunsuke Komoto, Takuya Komura, Naoki Konno, Yudai Koya, Koji Kubota, Mizuki Kuramochi, Hitoshi Kurata, Hideaki Kurihara, Michio Kuroki, Masaki Kuwatani, Takuro Maeda, Isamu Makino, Tomoo Manaka, Koichiro Mandai, Toru Maruo, Akinori Maruta, Yuichiro Maruyama, Yukiko Masuda, Takayuki Masuo, Saburo Matsubara, Satoshi Matsukuma, Hideo Matsumoto, Kazuyuki Matsumoto, Ryusuke Matsumoto, Yosuke Matsumura, Susumu Matsuo, Kisyo Mihara, Kei Mitsuhashi, Tsukasa Miyagahara, Ichiro Miyajima, Masaki Miyazawa, Shugo Mizuno, Suguru Mizuno, Kosuke Mori, Masayo Motoya, Shuntaro Mukai, Yoshiki Murakami, Yoshiyuki Murawaki, Yoko Murayama, Ryoji Nagamura, Hayato Nagase, Kazuyoshi Nagata, Kazunari Nakahara, Takaaki Nakahira, Yoshitaka Nakai, Yousuke Nakai, Akira Nakamura, Kazunori Nakaoka, Hiroshi Nakase, Atsushi Nakazawa, Yoshihito Nawa, Yusuke Niina, Tsutomu Nishida, Takayoshi Nishino, Shotaro Noge, Tatsuro Oaku, Tsuneyoshi Ogawa, Hideharu Ogiyama, Takaya Oguchi, Yoshito Ogura, Akihiko Ohata, Masahiro Ohtani, Hiroshi Ohyama, Hiromitsu Oka, Shiro Oka, Toshimasa Okada, Yasuyuki Okada, Hiromasa Okamoto, Kazuhisa Okamoto, Tomohiro Okamoto, Akihito Okazaki, Kazuichi Okazaki, Tetsuya Okino, Osamu Okochi, Fumihiro Okumura, Kosuke Okuwaki, Kiyohiro Oshima, Kazuhiro Otani, Takao Oyama, Akira Saeki, Kei Saito, Michihiro Saito, Nozomu Saito, Toshitaka Sakai, Yoshitaka Sakai, Misako Sakano, Takanori Sano, Yoshihiro Sasaki, Yuichi Sasakura, Masaaki Satake, Masahiro Sato, Satoshi Sato, Yoichiro Sato, Akihiko Satoh, Yugo Sawai, Masanari Sekine, Yasumasa Sekino, Morimichi Setsuda, Minoru Shigekawa, Masahiro Shiihara, Masaaki Shimatani, Takehiro Shimizu, Toru Shinkai, Nobuhiko Shinohara, Hideyuki Shiomi, Nakao Shirahata, Masayuki Sho, Yoshio Sogame, Takahiro Suda, Shigeyuki Suenaga, Hajime Sumi, Yasuyuki Sumida, Kaoru Suzuki, Keiichi Suzuki, Masato Suzuki, Noriaki Suzuki, Yuhei Suzuki, Kiyoshi Suzumura, Akinari Tabaru, Rei Takaesu, Kosuke Takagaki, Tadayuki Takagi, Kenichi Takahashi, Kenji Takahashi, Seiichi Takahashi, Shinichi Takano, Yuichi Takano, Yusuke Takasaki, Mamoru Takenaka, Hiroshi Tamagawa, Eiji Tamoto, Yu Tanaka, Shiroh Tanoue, Shuji Terai, Hiroaki Terajima, Masao Toki, Yuichi Torisu, Takayuki Tsujikawa, Keisuke Tsukamoto, Yuki Tsukamoto, Yosuke Tsuruga, Ken Tsushima, Hidehiko Tuda, Kazushige Uchida, Masayuki Ueno, Yoshiyuki Ueno, Jun Unno, Takahiro Urata, Sinnitirou Watanabe, Kenta Yamada, Reiko Yamada, Hirokazu Yamagami, Akira Yamamiya, Satoshi Yamamoto, Kohei Yamanouchi, Kentaro Yamao, Suguru Yamashita, Jun Yashika, Hiroaki Yasuda, Ichiro Yasuda, Tatsuji Yogi, Tomoyuki Yokota, Kentaro Yoneda, Hideo Yoshida, Hiroshi Yoshida, Naoki Yoshida, Hayato Yoshimura, Reiko Yoshioka

**Affiliations:** 1 Division of Gastroenterology, Tohoku University Graduate School of Medicine, 1-1 Seiryo-Machi, Aoba-ku, Sendai, 980-8574 Japan; 2 Department of Surgery, Faculty of Medicine, Kindai University, Osaka-Sayama, Japan

**Keywords:** Acute pancreatitis, Alcohol, ERCP, Necrosectomy, Step-up approach, Walled-off necrosis

## Abstract

**Objectives:**

This study aimed to clarify the current clinico-epidemiological characteristics of acute pancreatitis (AP) in Japan.

**Methods:**

We conducted a two-stage nationwide survey of patients with AP treated at selected hospitals in 2021, during the COVID-19 pandemic. The first stage estimated the total number of AP patients, while the second collected detailed clinical data.

**Results:**

The estimated number of AP patients requiring hospitalization was 61,080, with an overall incidence rate of 49 per 100,000 persons, decreasing from 78,450 in 2016. Detailed clinical data were obtained for 4,375 patients, including 1,362 (31.1%) classified as severe. The male-to-female ratio was 2.0, with mean ages at onset of 60.1 years for males and 65.4 years for females. The three major causes were alcohol (31.2%), gallstones (22.5%), and idiopathic etiology (22.1%). The AP-associated in-hospital mortality rate was 2.1% in all AP and 5.3% in severe cases, down from 6.1% in the 2016 survey. Antibiotics were administered to 61.2% of mild cases, a significant reduction from 94.5% in 2016. Enteral nutrition was provided to 56.9% of severe cases, up from 31.8% in 2016. Among 124 patients undergoing interventional drainage for walled-off necrosis, 57 were treated using a step-up approach. Notably, no patients underwent upfront surgery as the initial treatment.

**Conclusions:**

During the pandemic, the estimated number of AP cases requiring hospitalization declined for the first time in nearly four decades. Mortality in severe cases improved, and adherence to clinical guidelines on prophylactic antibiotics and enteral nutrition also improved, indicating enhanced management of AP in Japan.

**Supplementary Information:**

The online version contains supplementary material available at 10.1007/s00535-025-02284-2.

## Introduction

Acute pancreatitis (AP) is an acute inflammatory disease of the pancreas [1–3]. It primarily affects middle-aged and older individuals, with a global annual incidence of 33.7 cases per 100,000 persons in the general population, which has increased over the past few decades [4, 5]. Common causes of AP include alcohol, gallstones, idiopathic causes, and endoscopic retrograde cholangiopancreatography (ERCP) procedures [1–3]. While most patients experience a mild form of the disease, approximately 20% develop local complications, such as necrosis, and often systemic injury to major organs, including the cardiovascular, respiratory, and renal systems, due to systemic inflammation [6]. Morbidities resulting from local and systemic complications, as well as invasive interventions, have historically led to mortality rates as high as 30%. However, these rates have decreased over the past two decades, at least in part due to the implementation of clinical guidelines [1–4, 7, 8]. Several clinical guidelines for AP management have been published worldwide, including in Japan [9–12]. The Japanese guidelines were most recently revised in 2021 [12]; however, the impact of this revision on actual clinical practice remains unclear.

In Japan, nationwide epidemiological surveys have been conducted every 4–5 years since the 1980 s to clarify the clinical and epidemiological status of AP [13–18]. The estimated number of AP cases has continuously increased over the past three decades, reaching 78,450 in 2016, which represents a 25% increase compared to 2011 [17, 18]. Importantly, in-hospital mortality among patients with severe AP decreased markedly by 40%, from 10.1% in 2011 to 6.1% in 2016, suggesting improvements in the management of AP in Japan. These surveys have also provided insight into the implementation of clinical guidelines. The results of the nationwide epidemiological surveys have been incorporated into the revised guidelines, and changes have been made to enhance the compliance [8, 12, 18]. For example, despite strong recommendations, prophylactic antibiotic use was reported in 94.5% of patients with mild AP, while enteral nutrition (EN) was administered in only 31.8% of severe AP cases in the 2016 survey [18]. Based on these findings, the 2021 revision of the Japanese guidelines included modifications to specific clinical questions and recommendations [12]. Regarding the “Pancreatitis Bundles” (PBs), a set of clinical indicators defining key management measures, the item concerning prophylactic antibiotic use was revised to state that “no prophylactic antibiotics should be administered in mild AP,” and the recommendation for early EN was clarified to apply specifically to severe cases [12].

Real-world clinical data are essential for evaluating the current management of AP and refining clinical guidelines. To provide updated information on AP management in Japan, we conducted a nationwide epidemiological survey of patients with AP treated in 2021 during the coronavirus disease 2019 (COVID-19) pandemic [19].

## Methods

We conducted a two-stage survey. The first stage aimed to estimate the number of patients with AP, while the second stage focused on clarifying their clinical characteristics.

### Diagnosis of AP

The diagnosis of AP was made according to the clinical diagnostic criteria for AP [20], which require at least two of the following three conditions while excluding other pancreatic or acute abdominal diseases: (i) acute upper abdominal pain with tenderness; (ii) elevated levels of pancreatic enzymes in blood, urine, or ascitic fluid; and (iii) imaging findings of AP on ultrasonography, computed tomography (CT), or magnetic resonance imaging. Patients with AP were classified as having mild or severe disease according to the Japanese severity criteria proposed by the Ministry of Health, Labor and Welfare of Japan in 2008 (Japanese severity criteria) [21]. These criteria encompass nine prognostic factors and a contrast-enhanced CT grading system (Supplementary Table 1). We further classified the severe cases into “the most severe cases” fulfilling both the prognostic factor and CT grade criteria and “the remaining severe cases” fulfilling either the prognostic factor or CT grade criteria only.

### First-stage survey

Our target population comprised patients with AP who were treated at selected hospitals in 2021. These hospitals were chosen based on the Hospital Yearbook 2021 (R&D Co., Ltd., Nagoya, Japan). A list of critical care and emergency centers was obtained from the website of the Japanese Association for Acute Medicine (http://www.jaam.jp/html/shisetsu/qq-center.htm). Departments of internal medicine, gastroenterology, surgery, and digestive surgery were subjected to stratified random sampling. The sampling rates were approximately 5%, 10%, 20%, 40%, 80%, 100%, and 100% for hospitals with fewer than 100 beds, 100–199 beds, 200–299 beds, 300–399 beds, 400–499 beds, 500 or more beds, and university-affiliated hospitals, respectively. Critical care and emergency centers were treated as a special stratum, and all of them were included in the survey.

In July 2022, a questionnaire was mailed directly to 2,398 randomly selected departments as described above. The questionnaire inquired about the number of AP patients treated at their hospitals in 2021. After collecting the responses online, the number of AP patients, along with the 95% confidence interval (CI), was estimated under the assumption that department responses were independent of patient frequency, using the formulae described previously [22]. The population of Japan in 2021 (n = 125,502,000) (https://www.mhlw.go.jp/toukei/saikin/hw/jinkou/kakutei21/) was used to calculate the incidence rate.

### Second-stage survey

In July 2023, a second questionnaire was sent to departments that had reported treating AP patients in 2021 in response to the first questionnaire. The responses to the second questionnaire were collected online by the end of March 2024. Due to incomplete responses to certain items, the number of patients included in the analyses varied based on the specific item.

### Statistical analysis

Age distribution data are presented as mean ± standard deviation (SD). Cases with missing or unspecified values were excluded from the statistical analyses. Continuous variables were compared using Student’s *t* test, and proportions were compared using the chi-square test or Fisher’s exact test, as appropriate. The association between each prognostic factor and AP-related mortality was assessed using univariate logistic regression analysis. In addition, a multivariate logistic regression analysis was performed with all nine prognostic factors. To evaluate prognostic performance, a receiver operating characteristic (ROC) curve was generated, and the area under the curve (AUC) was calculated. All statistical analyses were performed using SPSS version 20.0 (SPSS Inc., Chicago, IL) or JMP Pro version 17 (SAS Institute, Cary, NC). A *P* value of < 0.05 was considered statistically significant.

## Results

### First-stage survey

**Fig. 1:**
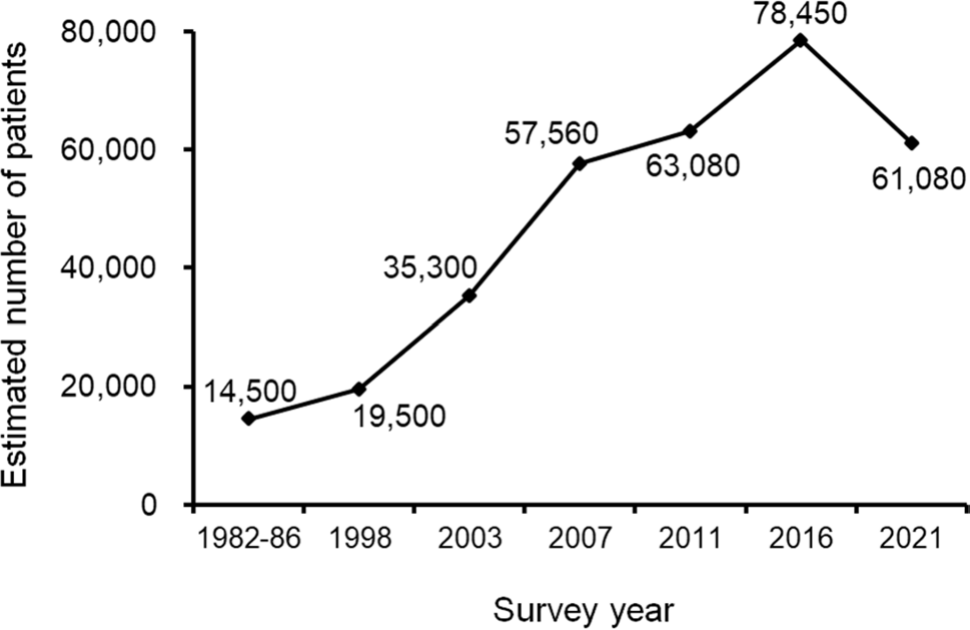
Trends in the estimated number of patients with AP since the first nationwide epidemiological survey

A total of 877 out of 2,398 departments responded to the first questionnaire, yielding a response rate of 36.6%. These departments reported 11,955 patients with AP (Supplementary Table 2). Based on this data, the estimated number of AP patients requiring hospitalization in 2021 was 61,080 (95% CI 57,220–64,940), corresponding to an annual incidence rate of 49 per 100,000 persons. Compared with the 2016 survey [18], this represents a 22% decrease. While the estimated number of AP patients had increased since the first survey, it declined for the first time in 2021 (Fig. 1).

### Second-stage survey

In the second-stage survey, detailed clinical information was collected for 4,375 patients with AP, including 2,903 males and 1,472 females, yielding a male-to-female ratio of 2.0. The overall mean age (SD) was 61.9 (18.4) years: 60.1 (17.5) years for males and 65.4 (19.5) years for females. The most frequently affected age group was 70–79 years for males and 80–89 years for females (Supplementary Fig. 1). Female patients were significantly older than male patients (*P* < 0.001).

### Initial symptoms

Supplementary Table 3 presents the initial symptoms reported by patients. The most common symptom was abdominal pain (92.5%), followed by vomiting (29.0%), back pain (22.0%), loss of appetite (21.8%), and fever (18.9%).

### Etiology

**Fig. 2:**
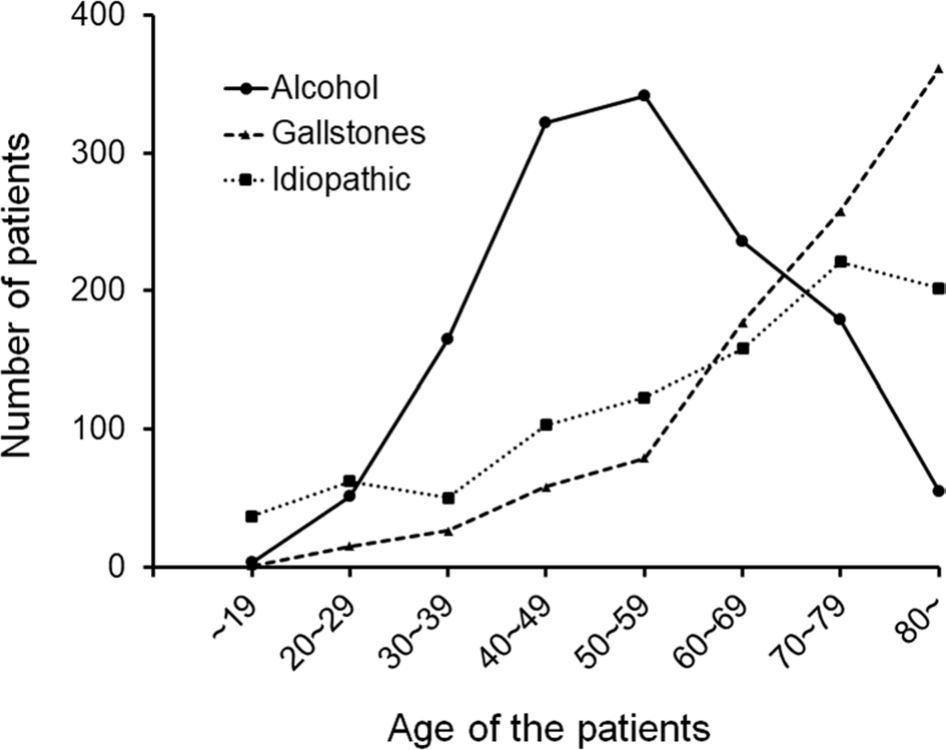
Age distribution of patients with AP, stratified by the three most common etiologies

Table 1 shows the etiology of AP. The three major causes for AP were Alcohol (31.2%), gallstones (22.5%), and idiopathic (22.1%) etiologies. The proportion of alcohol-related etiology was similar to that in the 2016 survey (32.6%). Alcohol was the leading cause (40.9%) among male patients, followed by idiopathic cause (18.6%) and gallstones (17.5%). Gallstones were the most common cause (32.4%) in female patients, followed by idiopathic cause (28.9%) and alcohol (12.2%). Alcohol-related AP was most frequently observed in individuals aged 50–59 years, while the incidence of gallstones and idiopathic pancreatitis cases increased with advancing age (Fig. 2). When stratified by sex and age, gallstone-induced AP was more prevalent among elderly female patients, alcohol-related AP was more common in middle-aged male patients, and the prevalence of idiopathic AP was similar between the sexes (Supplementary Fig. 2).

**Table 1:** Etiology of the enrolled patients with AP

Etiology	Male, n (%)	Female, n (%)	Total, n (%)
Alcohol	1,174 (40.9)	178 (12.2)	1,352 (31.2)
Gallstones	502 (17.5)	473 (32.4)	975 (22.5)
Idiopathic	534 (18.6)	422 (28.9)	956 (22.1)
Pancreatic tumor	139 (4.8)	75 (5.1)	214 (4.9)
Therapeutic ERCP	108 (3.8)	78 (5.3)	186 (4.3)
Hyperlipidemia	88 (3.1)	41 (2.8)	129 (3.0)
Surgery^*^	53 (1.9)	46 (3.2)	99 (2.3)
Immune checkpoint inhibitor-related	50 (1.7)	26 (1.8)	76 (1.8)
Diagnostic ERCP	40 (1.4)	21 (1.4)	61 (1.4)
Chronic pancreatitis	42 (1.5)	19 (1.3)	61 (1.4)
Pancreas divisum	27 (0.9)	12 (0.8)	39 (0.9)
Pancreaticobiliary maljunction	7 (0.2)	20 (1.4)	27 (0.6)
Duodenal papilla diseases	7 (0.2)	8 (0.6)	15 (0.3)
Drug^**^	8 (0.3)	5 (0.3)	13 (0.3)
Autoimmune pancreatitis	12 (0.4)	1 (0.1)	13 (0.3)
Other endoscopic procedures^***^	8 (0.3)	3 (0.2)	11 (0.3)
Hereditary/Familial	8 (0.3)	2 (0.1)	10 (0.2)
COVID-19	10 (0.4)	0 (0)	10 (0.2)
Abdominal injury	4 (0.1)	2 (0.1)	6 (0.1)
Others	51 (1.8)	28 (1.9)	79 (1.8)
Total	2,872 (100)	1,460 (100)	4,332 (100)

Etiology of AP was not described in 31 males and 12 females

^*^Includes cases due to the stenosis of pancreatic anastomosis

^**^Excludes cases with immune checkpoint inhibitor-related AP

^***^Includes cases related to endoscopic ultrasonography-fine needle aspiration

AP, acute pancreatitis; COVID-19, coronavirus disease 2019; ERCP, endoscopic retrograde cholangiopancreatography

### Severity

Based on the Japanese severity criteria [21], 3,013 patients were classified as mild (68.9%; 1,976 males and 1,037 females), while 1,362 patients were classified as severe (31.1%; 927 males and 435 females). Considering the total number and proportion of severe cases, the estimated number of patients with mild and severe AP was 42,080 and 19,000, respectively. Compared to the 2016 survey, the estimated number of patients with mild AP decreased by 29.8%, from 59,940, while the number for severe AP remained similar to 2016 (18,510).

Among the 1,362 patients with severe AP, 1,022 (75.0%) were classified as severe based solely on CT grade, 152 (11.2%) solely on prognostic factors, and 188 (13.8%) on both CT grade and prognostic factors. The mean (SD) age of patients with severe AP was 60.4 (19.3) years: 57.8 (18.4) for males and 65.9 (19.9) for females. The distribution of patients with severe AP stratified by age and sex is shown in Supplementary Fig. 3. The most affected age group was 70–79 years for both male and female patients. Alcohol was the most common cause (37.3%), followed by idiopathic cause (22.3%) and gallstones (21.5%) (Supplementary Table 4).

### Mortality rate

**Fig. 3:**
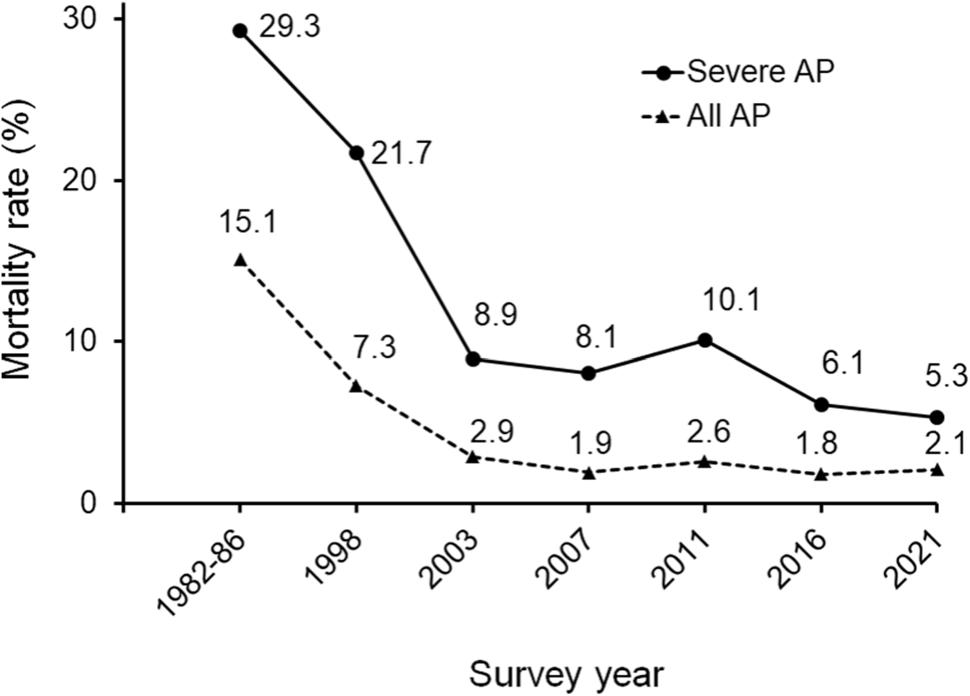
Trends in mortality rates among all patients with AP and those with severe AP since the first nationwide epidemiological survey

Among the 4,375 patients, 90 (2.1%) died from AP. Forty patients died within 2 weeks of admission, while the remaining 50 died thereafter. In severe AP cases, 31 patients died within 2 weeks and 41 died after 2 weeks. The mortality rate was 0.6% in mild AP cases and 5.3% in severe AP cases. Figure 3 illustrates the trends in mortality rates among patients with AP since the first nationwide epidemiological survey. Compared to the 2016 survey, the mortality rate in patients with severe AP decreased by 13.1%, from 6.1% to 5.3%. When stratified by etiology, the mortality rate in patients with AP related to therapeutic ERCP was 4.8% (9/186), which was higher than that of alcohol-related (1.8%, 25/1,352) (*P* = 0.009), gallstone-related (2.1%, 20/975) (*P* = 0.03), and idiopathic (2.1%, 20/956) cases (*P* = 0.03).

The mortality rate was 13.2% in patients classified as severe who met only the prognostic factors, 2.4% in those who met the CT grade alone, and 14.4% in the most severe cases fulfilling both the prognostic factors and the CT grade (Supplementary Table 5). The mortality rate of the remaining severe cases was 3.8% (45/1,174). The mortality rate of patients meeting the CT grade criteria (4.3%) was lower than that of patients meeting the prognostic scores (13.8%) (*P* < 0.001).

The mortality rate increased with the number of positive prognostic factors (Supplementary Fig. 4). The mortality rate of patients with prognostic scores less than 2 was 1.1% (43/4,035), whereas that of patients with scores of 3 or higher was 13.8% (47/340). Among patients with prognostic factor scores of 6 or higher, the mortality rate was as high as 34.7% (17/49). We performed ROC curve analysis to evaluate the predictive accuracy of the prognostic factor scores for mortality. The AUC for predicting mortality was 0.84. If the cutoff point was set at a prognostic factor score of 2, as currently defined in the severity criteria [21], the sensitivity reached 0.76 with a specificity of 0.81. We analyzed which prognostic factors were most closely associated with mortality. All nine prognostic factors were associated with AP-related death by univariate analysis (all *P* < 0.0001) (Table 2). A multivariate analysis identified six out of the nine prognostic factors (base excess ≤ −3 mEq/L or shock, PaO_2_ ≤ 60 mmHg or respiratory failure, elevated lactate dehydrogenase, platelet count ≤ 100,000/μL, serum calcium ≤ 7.5 mg/dL, and age ≥ 70 years) that were associated with AP-related death. Among these, age ≥ 70 years had the highest odds ratio.

**Table 2:** Univariate and multivariate analyses showing the association of prognostic factors with AP-related death

	Univariate analysis			Multivariate analysis	
	OR (95% CI)	*P* value		OR (95% CI)	*P* value
Base excess ≤ −3 mEq/L or shock	11.7 (7.6–18.1)	< 0.0001		4.3 (2.4–7.7)	< 0.0001
PaO_2_ ≤ 60 mmHg or respiratory failure	12.4 (7.4–20.8)	< 0.0001		2.2 (1.1–4.5)	0.03
BUN ≥ 40 mg/dL (or Cr ≥ 2 mg/dL) or oliguria	7.8 (4.9–12.4)	< 0.0001		1.7 (0.9–3.1)	0.09
Elevation of LDH (twice or more than UNL)	6.3 (4.1–9.6)	< 0.0001		2.0 (1.2–3.5)	0.01
Platelet count ≤ 100,000/μL	6.7 (3.9–11.6)	< 0.0001		2.1 (1.1–4.1)	0.04
Serum calcium ≤ 7.5 mg/dL	15.1 (9.1–25.0)	< 0.0001		3.6 (1.7–7.6)	0.0006
CRP ≥ 15 mg/dL	3.2 (2.0–5.0)	< 0.0001		0.9 (0.5–1.7)	0.69
SIRS criteria ≥ 3	7.2 (4.7–11.2)	< 0.0001		1.6 (0.8–3.0)	0.16
Age ≥ 70 years	3.7 (2.4–5.9)	< 0.0001		5.3 (3.1–8.8)	< 0.0001

AP, acute pancreatitis; BUN, blood urea nitrogen; CI, confidence interval, CRP, C-reactive protein; LDH, lactate dehydrogenase; OR, odds ratio; SIRS; systemic inflammatory response syndrome

### Management

#### Initial fluid resuscitation

The total volume of fluid resuscitation administered during the first 24 h after admission was reported in 3,947 cases (2,688 mild and 1,259 severe). The mean volume (SD) was 2,593 (1,221) mL in mild AP and 3,239 (1,640) mL in severe AP cases. The volume was greater in severe AP cases than in mild cases (*P* < 0.001). Among the severe cases, the volume was 3,484 (3,170) mL in fatal cases (n = 68) and 3,225 (1,508) mL in survived cases (n = 1,191). The volume was not different between the fatal and survived cases (*P* = 0.21).

#### Enteral nutrition

Information about EN was available in 3,980 cases (2,675 mild and 1,305 severe cases, including 183 most severe cases). Eight hundred sixty-six (32.4%) mild cases and 743 (56.9%) severe cases received EN. Among the 1,305 severe cases, 133 out of 183 (72.7%) cases with the most severe AP and 610 out of the remaining 1,122 (54.4%) severe cases received EN. The proportion of patients who received EN was higher in those with the most severe AP compared to other severe cases and those with mild AP (*P* < 0.001). The timing of EN initiation was reported in 819 mild and 727 severe AP cases (Supplementary Table 6). Supplementary Fig. 4 illustrates the proportions of patients with severe AP who received EN, stratified by the timing of its initiation in the 2021 and 2016 surveys. In the 2021 survey, EN was initiated within 24 and 48 h of admission in 12.1% and 23.2% of patients, respectively. In contrast, in the 2016 survey, only 1.9% and 5.9% of patients with severe AP received EN within 24 and 48 h, respectively.

#### Prophylactic antibiotics

Antibiotics were administered to 61.2% (1,786/2,918) mild and 82.9% (1,114/1,344) severe AP cases, primarily for prophylactic purposes. The cephem and β-lactamase inhibitor combination was most frequently used in mild cases, while carbapenem was used in severe AP cases (Supplementary Table 7).

#### Management of walled-off necrosis

Information about walled-off necrosis (WON) was available in 3,932 patients; WON developed in 365 (9.3%) of these cases. A comparison of the clinical characteristics of patients with WON and those without WON revealed that patients with WON were predominantly male and had more severe disease, as indicated by a higher number of positive prognostic factors and higher CT grade scores (Table 3). The mortality rate was higher in patients with WON (4.9%) compared to those without WON (1.4%).

**Table 3:** Comparison of clinical characteristics between the patients with WON and those without it

	WON (+) n = 365	WON (–) n = 3,562	*P* value
Sex, male, n (%)	280 (76.7)	2,317 (65.1)	< 0.001
Age, years, mean (SD)	60.1 (16.6)	62.1 (18.5)	0.04
Severe case, n (%)	247 (67.7)	967 (27.2)	< 0.001
Hypo-enhanced lesion score on CT^#^, mean (SD)	0.32 (0.67)	0.05 (0.25)	< 0.001
CT grade score^#^, mean (SD)	1.71 (1.10)	0.74 (0.91)	< 0.001
Number of positive prognostic factors^#^, mean (SD)	1.64 (1.71)	0.84 (1.08)	< 0.001
Fatal case, n (%)	18 (4.9)	48 (1.3)	< 0.001

^#^: According to the Japanese severity classification system

CT, computed tomography; SD, standard deviation; WON, walled-off necrosis

**Fig. 4:**
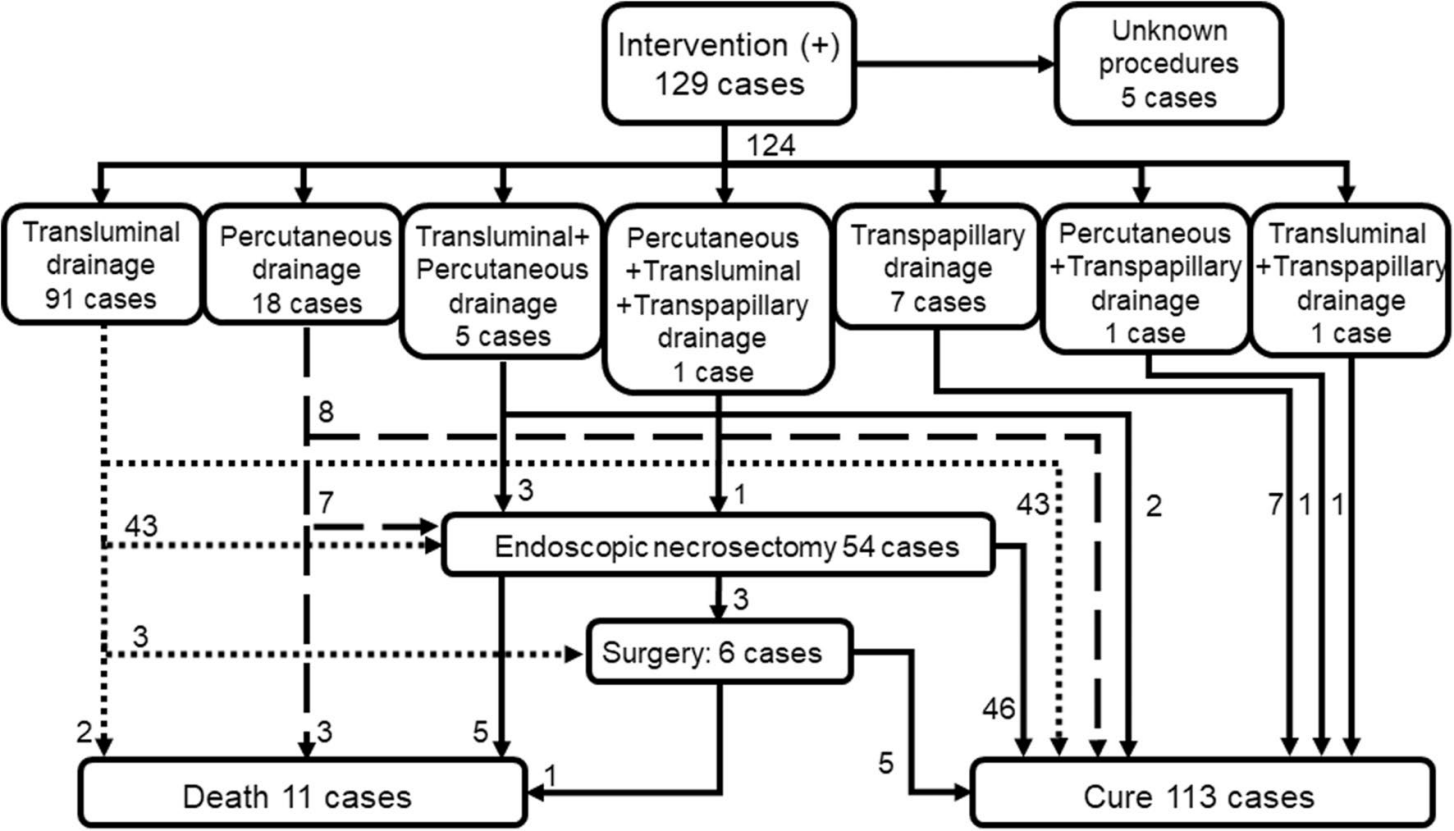
Flow chart of interventions for WON

Information about the therapeutic intervention for WON was available for 365 patients. One hundred twenty-nine (35.3%) patients underwent intervention for WON; however, detailed intervention procedures were not provided for five patients, one of whom died. No patients received upfront surgery as an initial treatment, and 124 patients received drainage through transluminal, percutaneous, and/or transpapillary routes, either individually or in combination (Fig. 4). Sixty-one (49.2%) patients were cured solely by drainage therapies, but six patients died of AP. The remaining 57 patients underwent a step-up approach after drainage: endoscopic necrosectomy alone (n = 51), surgery alone (n = 3), and both (n = 3). Fifty-two patients were cured, but five patients died. In total, 12 out of 129 (9.3%) patients who received interventions and 7 out of 236 (3.0%) patients who received conservative treatment died (*P* = 0.009).

## Discussion

We conducted a nationwide epidemiological survey to clarify the current status of AP in Japan. The estimated number of AP patients requiring hospitalization in 2021 was 61,080, representing a 22% decrease compared to the 2016 survey—the first significant decline in nearly four decades of nationwide epidemiological surveys in Japan [13–18]. This decrease was primarily attributed to a 29.8% reduction in mild AP cases, while the number of patients with severe AP increased by 2.6%. The COVID-19 pandemic, which was ongoing in 2021, was likely a major factor contributing to the decline in mild AP cases. The pandemic placed a substantial burden on the healthcare system, affecting not only resources directly used for treating COVID-19 but also those required for managing other serious conditions such as AP [23–28]. In Japan, urgent hospital admissions at acute care hospitals decreased by 10.4% between February and December 2020 compared to the same period in 2019, according to the Diagnosis Procedure Combination database [24].

At the onset of the pandemic, an increased incidence of alcohol-related AP, possibly due to higher alcohol consumption during lockdowns [29], was reported in Japan [30]. However, many subsequent studies from around the world indicated a decline in hospital admissions for AP during the pandemic [25–28]. Du et al. [25] found a 39% reduction in AP admissions in 2020 at a regional medical center in northeastern China. Ramsey et al. [26] reported a 15% decrease in AP admissions at the Ohio State University Wexner Medical Center in the United States over the same period. Ngu et al. [27] observed a 22% reduction in pancreatitis admissions at the largest tertiary health service in Victoria, Australia, from 2019 to 2020, which persisted into 2021. In addition, Adekunle et al. [28] reported an 11% decline in total hospital discharges between 2019 and 2020 using data from the California State Inpatient Database. Possible explanations for these reductions include patients’ reluctance to seek medical care—particularly for mild symptoms—due to fear of exposure to the virus, and hospitals'hesitancy to admit non-critical AP cases amid resource constraints. This hypothesis is supported by delayed hospital presentations, which have been associated with increased disease severity, higher rates of pancreatic necrosis, systemic inflammatory response syndrome, and persistent organ failure compared to the pre-pandemic period [25–28]. In line with these global observations, the estimated number of patients with mild AP in our 2021 survey decreased by nearly 30%, while the proportions of patients with severe AP and those with WON increased to 31.1% and 9.3%, respectively, compared to 23.6% and 6.9% in the 2016 survey. Our findings suggest that a similar reluctance to seek hospital care and a higher admission threshold for mild AP cases also occurred in Japan during the pandemic.

The overall mortality rate among patients with AP increased from 1.8% in 2016 to 2.1% in 2021, reflecting a higher proportion of severe cases. A similar trend was observed in the United States. Lo et al. [31] found that the age-adjusted AP-related mortality decreased from 3.31 deaths per 100,000 population in 1999 to 2.25 in 2019, but rose to 2.72 in 2020 during the COVID-19 pandemic. Chaudhry et al. [32] reported an increase in mortality among patients with alcohol-related pancreatitis: a 48% increase in males and a 64% increase in females in 2021 compared to 2018. Importantly, in this study, the mortality rate among severe AP cases improved from 6.1% in 2016 to 5.3% in 2021. Given that the same severity criteria have been applied in nationwide surveys since 2011, it is reasonable to attribute this improvement to better management of AP. Our findings suggest that inpatient care for AP remained effective and timely, even under the constraints imposed by the pandemic. Nevertheless, attention should be paid to the relatively high mortality rate among patients with therapeutic ERCP-related AP, as Japan is a rapidly aging country and elderly patients frequently undergo ERCP for the treatment of gallstones and pancreatobiliary tumors [33, 34].

In Japan, the severity classification system defines AP as either mild or severe, while other well-known classifications, such as the revised Atlanta classification [35], categorize AP into three grades: mild, moderately severe, and severe. In this study, we further stratified the Japanese “severe” AP cases by defining those who met both the prognostic score and CT grade criteria as having “most severe AP,” with a mortality rate of 14.4%. In contrast, the remaining severe cases had a mortality rate of 3.8%. Given that the overall mortality among patients with severe AP has now declined to approximately 5%, it may be appropriate to begin discussions about revising the current Japanese severity classification system to better reflect clinical outcomes and align with international standards.

The mortality rates among patients with AP decreased both during the first 2 weeks of admission (from 2.7% to 2.3%) and after 2 weeks (from 3.4% to 3.0%). The 2016 survey revealed a significant reduction in mortality within the first 2 weeks, but the mortality rate after this period remained unchanged. One possible explanation for the reduction in mortality after 2 weeks is the improved management of WON, including the coverage of lumen-apposing metal stents by the national health insurance in 2018 [36]. Over the past two decades, a paradigm shift has occurred in the treatment of WON [37–40]. The minimally invasive step-up approach has demonstrated superiority over open necrosectomy, while endoscopic access is more effective than percutaneous surgical approaches in reducing the incidence of new-onset multiorgan failure, the duration of hospital stays, late fistula formation, and overall treatment costs [37–40]. Currently, endoscopic ultrasound-guided drainage serves as a first-line treatment for symptomatic WON at many centers. This shift is reflected in the nationwide epidemiological surveys in Japan; the proportion of patients with WON undergoing upfront surgery has dramatically decreased over the past two decades—from 33.3% in 2007 to 22.5% in 2011, 4.7% in 2016, and 0% in 2021 [18, 41]. Despite this evolution in clinical practice, mortality rates among patients with WON have not significantly improved across the last three national surveys: 5.9% (7/118) in 2011, 6.7% (13/195) in 2016, and 4.9% (18/365) in 2021 (*P* = 0.39 for the comparison between 2016 and 2021 surveys). Similarly, among patients who underwent interventions, mortality rates remained relatively unchanged: 7.7% (3/39) in 2011, 9.3% (10/107) in 2016, and 9.3% (12/129) in 2021. These results strongly suggest that there is room for improvement in the management of WON, including the indications and timing of interventions, standardization of endoscopic procedures, and long-term management [42]. Recently, Hamada et al. [43] reported that a higher Charlson’s Comorbidity Index was associated with an increase in in-hospital mortality among patients undergoing endoscopic ultrasound-guided drainage of pancreatic fluid collections. Proper periprocedural mortality risk stratification will be essential for further reducing late-phase mortality.

One of the major aims of the nationwide epidemiological survey is to evaluate compliance with clinical guidelines. International surveys have demonstrated that the most common discrepancy between daily clinical practice and recommendations included the use of prophylactic antibiotics and early enteral feeding [44, 45]. Indeed, previous nationwide surveys in Japan revealed significant discrepancies between the guideline recommendations and actual clinical practice, particularly regarding early EN and prophylactic antibiotics [18, 41]. To fill these gaps, the 2021 revision of the Japanese guidelines for AP included modifications to the PBs items. In this study, the proportion of patients with severe AP who received EN nearly doubled, from 31.8% in 2016 to 56.9% in 2021. Early initiation of EN within 48 h of AP diagnosis was achieved in 18.6% of cases undergoing EN in 2016, but rose markedly to 40.7% of these cases in 2021. Notably, the proportion of patients receiving EN increased with the level of severity, suggesting that the importance of early EN based on the disease severity has been recognized in daily clinical practice. Similarly, although the guidelines discourage prophylactic antibiotic use in mild AP, 94.5% of patients with mild AP received antibiotics in 2016. While this figure remained high, it declined substantially to 61.2% in 2021. Because adherence to PB items is associated with reduced mortality in patients with severe AP [8], further compliance with these recommendations is expected to contribute to better clinical outcomes.

This study has several limitations. First, it was a retrospective, responder-based survey that utilized available medical records, which may have introduced selection bias and resulted in missing data. Second, the cases included in the second survey may not fully represent the estimated 61,080 cases of AP nationwide, as data from small hospitals and emergency centers were limited due to relatively low sampling and response rates. However, a previous study demonstrated that the characteristics of AP patients identified in a nationwide survey were comparable to those reported in the Diagnosis Procedure Combination database [46]. Third, this survey focused exclusively on inpatients, and outpatients were not included. Although the Japanese guidelines recommend hospitalization for all patients with AP [12], both physicians and patients were likely hesitant to pursue admission during the COVID-19 pandemic due to concerns about virus exposure and the strain on hospital resources. Despite these limitations, this study has several strengths. First, by employing the same methodological framework as previous nationwide epidemiological surveys, we were able to assess long-term trends in the estimated number of AP cases over four decades. This consistency also enabled meaningful comparisons of clinical practices over time, including trends in fluid resuscitation, EN, and antibiotic use. Second, detailed clinical data were collected from over 4,000 AP patients treated across Japan within a single year, providing a comprehensive snapshot of real-world clinical practice. Third, this is the first nationwide survey to comprehensively assess the impact of the COVID-19 pandemic—a unique and unprecedented event—on the clinical management of AP in Japan.

## Conclusions

This nationwide survey revealed, for the first time in nearly four decades, a decrease in the estimated number of patients with AP, primarily due to a reduction in hospitalizations for mild cases during the COVID-19 pandemic. The mortality rate for severe AP continued to decline. Importantly, although adherence to clinical guidelines—particularly regarding prophylactic antibiotics and early EN—has significantly improved, it remains suboptimal. Moreover, the mortality rate among patients undergoing interventions for WON has largely remained unchanged, indicating that further improvements in the management of AP are needed. We believe that the up-to-date insights provided by this study will contribute to further improving the prognosis of patients with this intractable disease.

## Supplementary Information

Below is the link to the electronic supplementary material.

Supplementary file1 (DOCX 247 KB)

Supplementary file2 (DOCX 38 KB)

## Abbreviations

AP: Acute pancreatitis
AUC: Area under the curve
CI: Confidence interval
COVID-19: Coronavirus disease 2019
CT: Computed tomography
EN: Enteral nutrition
ERCP: Endoscopic retrograde cholangiopancreatography
PB: Pancreatitis Bundles
ROC: Receiver-operating characteristic
SD: Standard deviation
WON: Walled-off necrosis
