# Supplementary material for: Nationwide epidemiological survey of acute pancreatitis in Japan, 2021: the impact of the COVID-19 pandemic and revised clinical guidelines
Source: J Gastroenterol. 2025 Jul 31;60(11):1437–48. doi: 10.1007/s00535-025-02284-2 (PMC12549737; doi:10.1007/s00535-025-02284-2)
Supplement: Supplementary file 1 — Supplementary file1 (DOCX 247 KB) [file 535_2025_2284_MOESM1_ESM.docx]

**
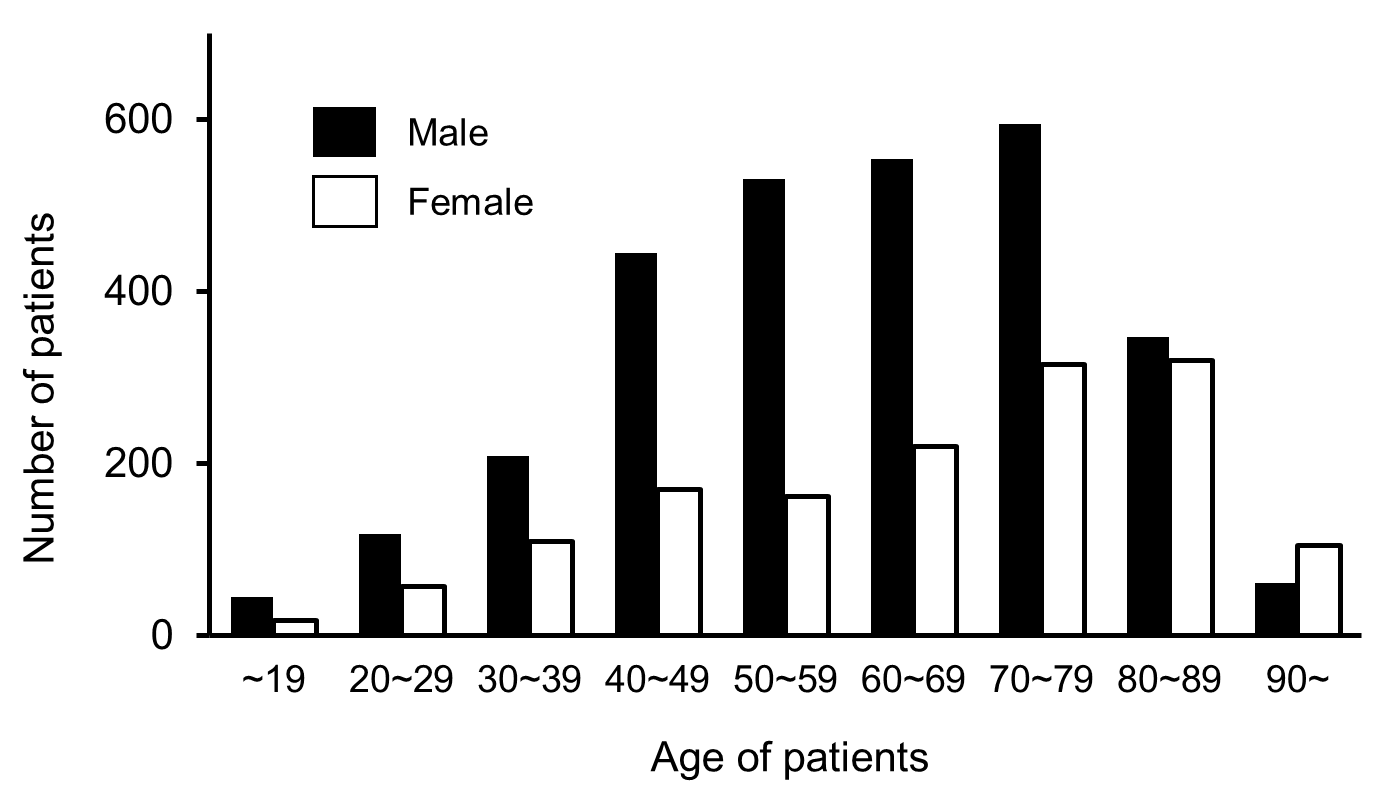
**

**Supplementary Figure 1. Age distribution of patients with acute pancreatitis, stratified by sex.**

**
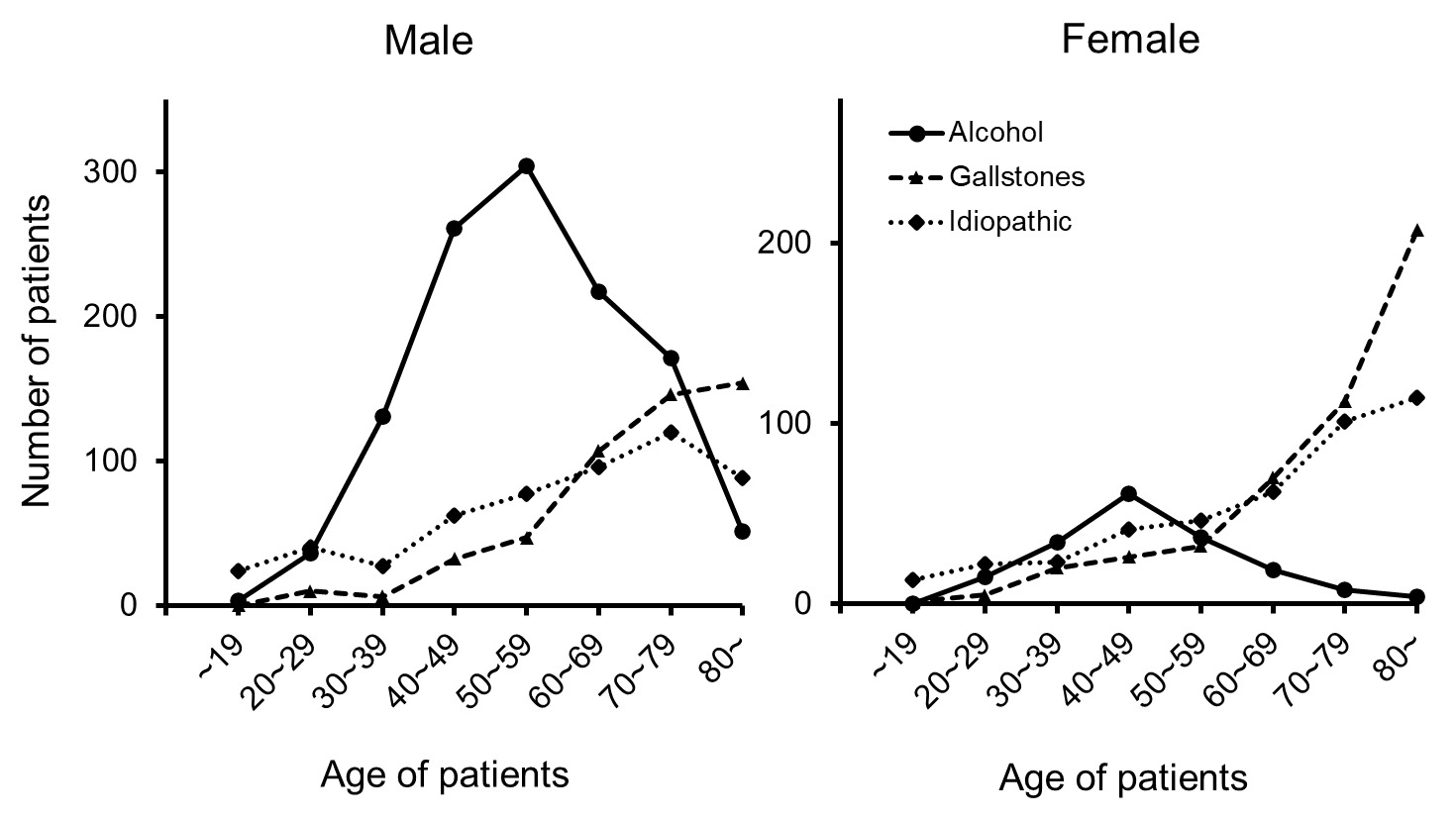
**

**Supplementary Figure 2. Age distribution of patients with acute pancreatitis, stratified by sex and the three most common etiologies.**

**
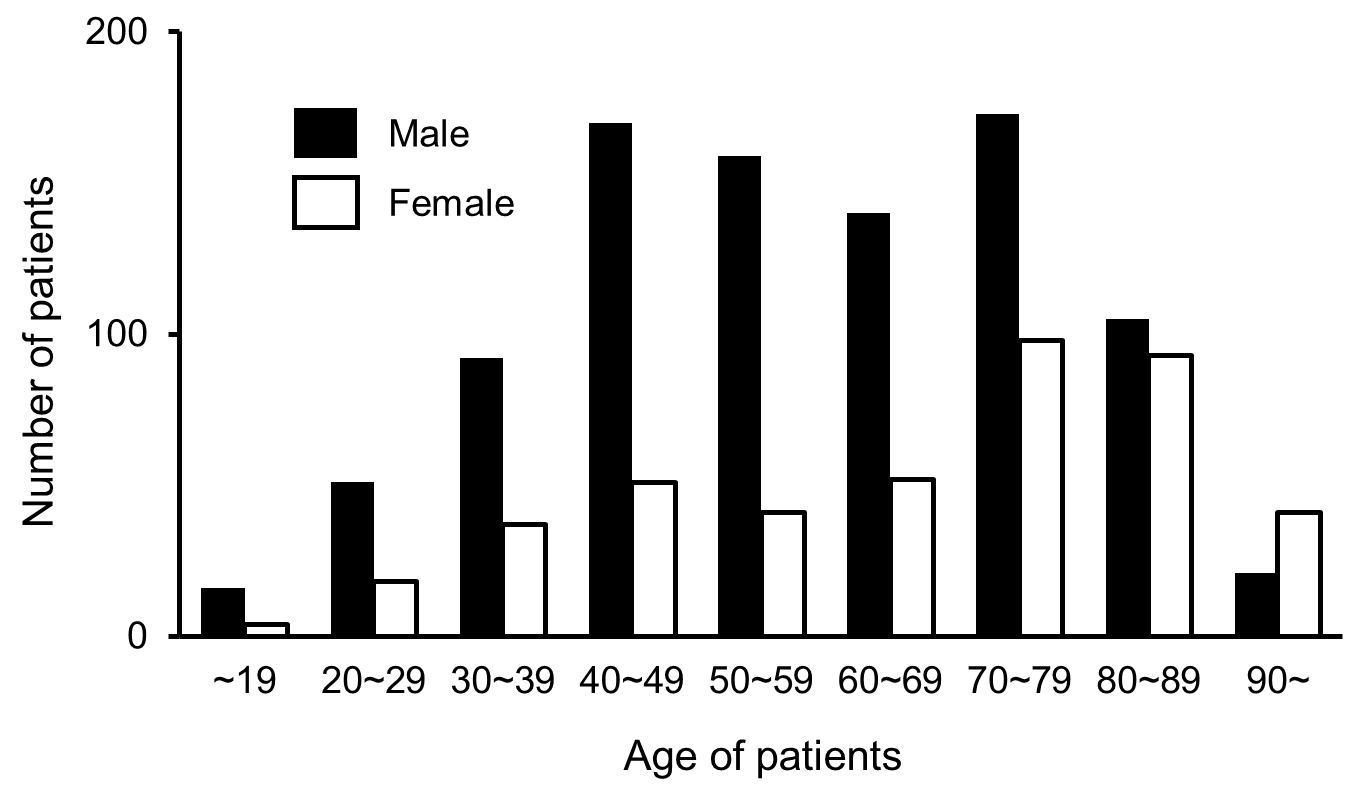
**

**Supplementary Figure 3. Age distribution of patients with severe acute pancreatitis, stratified by sex.**

**
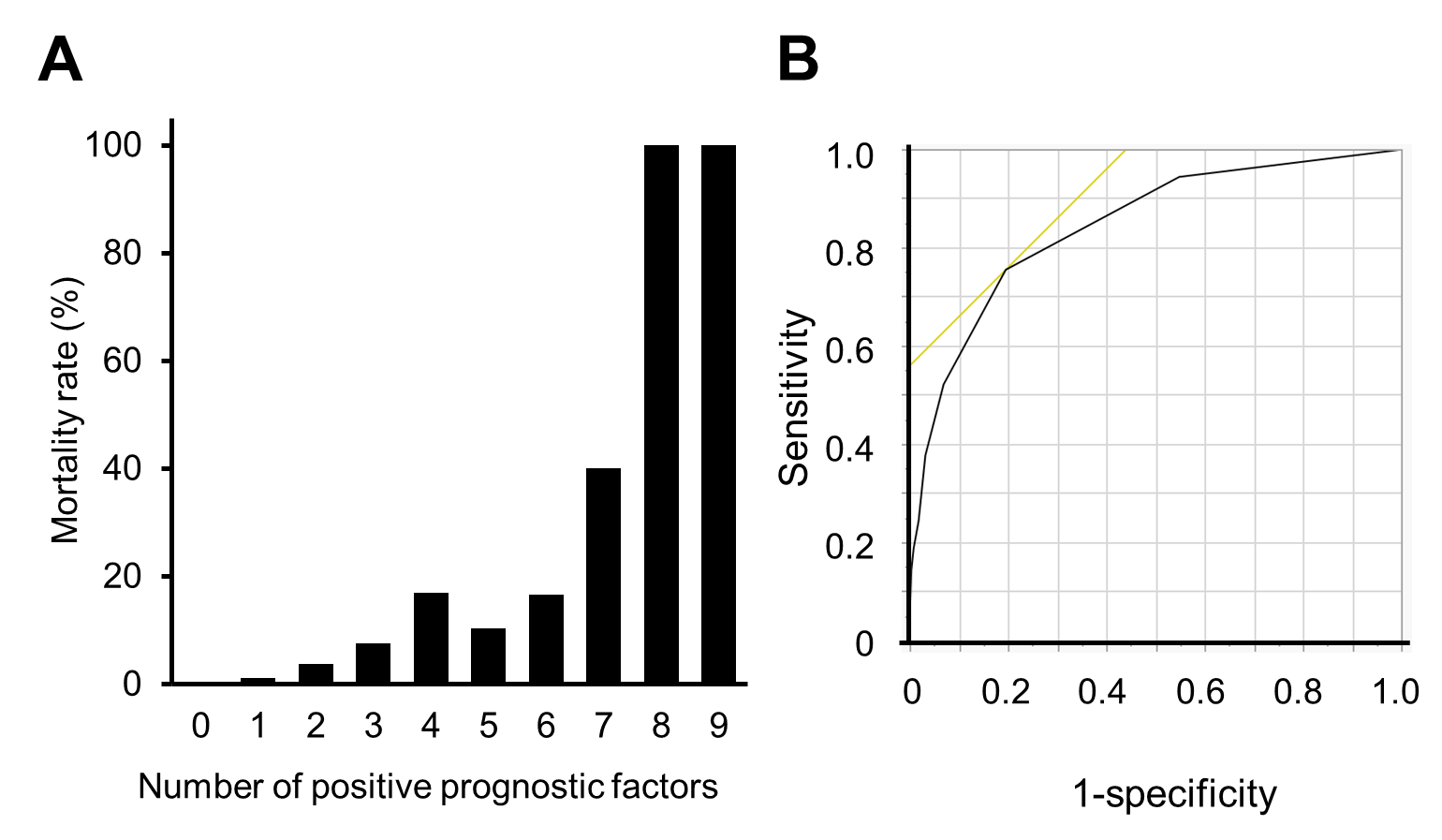
**

**Supplementary Figure 4. Performance of prognostic factors to predict mortality.**

**(A)** Mortality of the patients with acute pancreatitis according to the number of positive prognostic factors. (**B**) Receiver-operating characteristics curve analysis of the nine prognostic factor items. The area under the curve for predicting mortality was 0.84.

**
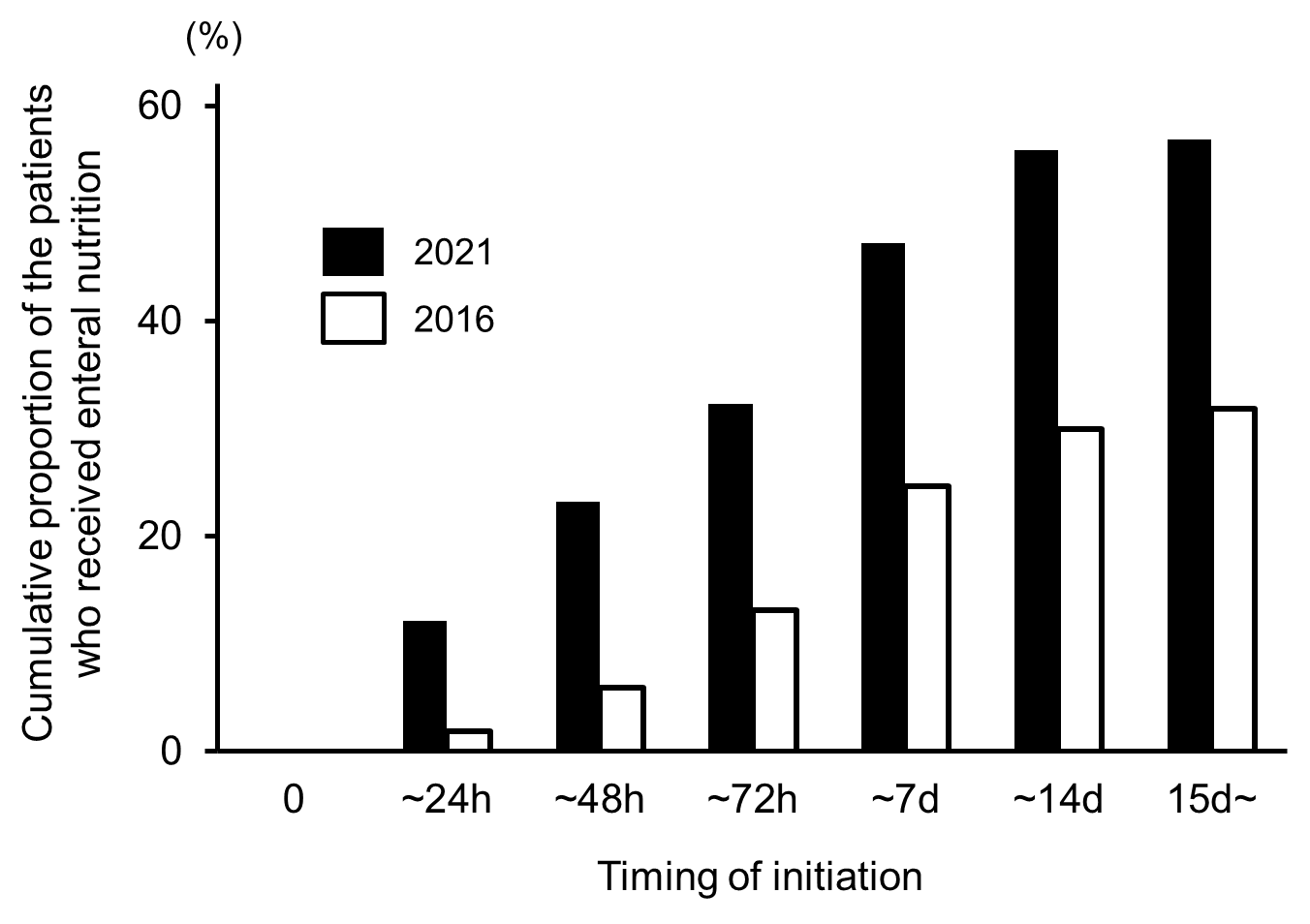
**

**Supplementary Figure 5. Cumulative proportions of patients with severe acute pancreatitis who received enteral nutrition, stratified by the timing of its initiation, in the 2021 and 2016 surveys.**

The proportions were calculated based on the number of patients who received enteral nutrition and the timing of its initiation among those with available data. In the 2021 survey, enteral nutrition was initiated within 24 hours (h), 48 h, 72 h, 7 days (d), 14 d, and ≥15 d in 12.1%, 23.2%, 32.3%, 47.2%, 55.9%, and 56.9% of patients with severe acute pancreatitis, respectively. In the 2016 survey, the corresponding proportions were 1.9%, 5.9%, 13.1%, 24.6%, 29.9%, and 31.8%.
