# Supplementary material for: Nationwide epidemiological survey of acute pancreatitis in Japan, 2021: the impact of the COVID-19 pandemic and revised clinical guidelines
Source: J Gastroenterol. 2025 Jul 31;60(11):1437–48. doi: 10.1007/s00535-025-02284-2 (PMC12549737; doi:10.1007/s00535-025-02284-2)
Supplement: Supplementary file 2 — Supplementary file2 (DOCX 38 KB) [file 535_2025_2284_MOESM2_ESM.docx]

| **Supplementary Table 1. The severity criteria for acute pancreatitis proposed by the Ministry of Health, Labour and Welfare of Japan in 2008** | | | |
| --- | --- | --- | --- |
| **Prognostic factors (1 point for each factor)** | | | |
|  | 1. | Base excess ≤ -3 mEq/L or shock (systolic blood pressure < 80 mmHg) | |
|  | 2. | PaO_2_ ≤ 60 mmHg (room air) or respiratory failure (respirator management is needed) | |
|  | 3. | BUN ≥ 40 mg/dL (or Cr ≥ 2 mg/dL) or oliguria (<400 ml/day even after fluid therapy) | |
|  | 4. | LDH ≥ 2 times of upper limit of normal | |
|  | 5. | Platelet count ≤ 100,000/μL | |
|  | 6. | Serum calcium ≤ 7.5 mg/dL | |
|  | 7. | CRP ≥ 15 mg/dL | |
|  | 8. | Number of positive measures in SIRS criteria ≥ 3 | |
|  | 9. | Age ≥ 70 years | |
|  |  |  | |
| **CT grade by contrast-enhanced CT** | | | |
|  | 1. | Extrapancreatic progression of inflammation | |
|  |  | Anterior pararenal space | 0 point |
|  |  | Root of mesocolon | 1 point |
|  |  | Beyond the lower pole of the kidney | 2 points |
|  | 2. | Hypo-enhanced region of the pancreas | |
|  |  | The pancreas is divided into three segments: head, body, and tail | |
|  |  | Localized within each segment or only surrounding the pancreas | 0 point |
|  |  | Covers two segments | 1 point |
|  |  | Occupies the entire two segments or more | 2 points |
|  | 1+2 = Total scores | | |
|  |  | Total score = 0 or 1 | Grade 1 |
|  |  | Total score = 2 | Grade 2 |
|  |  | Total score =3 or more | Grade 3 |
| (1) If prognostic factors are scored as 3 points or more, or (2) if CT Grade is judged as two or more, the severity grading is evaluated to be as ‘‘severe’’.  AP, acute pancreatitis; BUN, blood urea nitrogen; CRP, C-reactive protein; CT, computed tomography; LDH, lactate dehydrogenase; SIRS; systemic inflammatory response syndrome | | | |

| **Supplementary Table 2. Summary of the first-stage survey** | | | | | |
| --- | --- | --- | --- | --- | --- |
| Strata | Total number of departments | Sampling rate (%) | Number of surveyed departments | Number of responding departments | Response  rate (%) |
| University　hospital | 250 | 100 | 250 | 187 | 74.8 |
| Emergency unit | 299 | 100 | 299 | 51 | 17.1 |
| > 500 beds | 514 | 100 | 514 | 212 | 41.3 |
| 400–499 beds | 495 | 80 | 396 | 155 | 39.1 |
| 300–399 beds | 855 | 40 | 342 | 98 | 28.7 |
| 200–299 beds | 862 | 20 | 172 | 65 | 37.8 |
| 100–199 beds | 2,867 | 10 | 286 | 77 | 26.9 |
| < 99 beds | 2,785 | 5 | 139 | 32 | 23.0 |
| Total | 8,927 | - | 2,398 | 877 | 36.6 |

| **Supplementary Table 3. Initial symptoms** | |
| --- | --- |
| Symptoms* | n (%) |
| Abdominal pain | 4,049 (92.5) |
| Vomiting | 1,269 (29.0) |
| Back pain | 963 (22.0) |
| Appetite loss | 955 (21.8) |
| Fever | 825 (18.9) |
| Abdominal distention | 683 (15.6) |
| General fatigue | 600 (13.7) |
| Jaundice | 365 (8.3) |
| Diarrhea | 216 (4.9) |
| Conscious disturbance | 79 (1.8) |
| Shock | 73 (1.7) |
| Others | 76 (1.7) |
| Total | 4,375 (100) |
| *including overlapping symptoms | |

| **Supplementary Table 4. Etiology of severe AP cases** | | | |
| --- | --- | --- | --- |
| Etiology | Male, n (%) | Female, n (%) | Total, n (%) |
| Alcohol | 424 (46.0) | 79 (18.5) | 503 (37.3) |
| Idiopathic | 172 (18.7) | 128 (29.9) | 300 (22.3) |
| Gallstones | 154 (16.7) | 136 (31.8) | 290 (21.5) |
| Hyperlipidemia | 34 (3.7) | 13 (3.0) | 47 (3.5) |
| Therapeutic ERCP | 27 (2.9) | 16 (3.7) | 43 (3.2) |
| Pancreatic tumor | 24 (2.6) | 13 (3.0) | 37 (2.7) |
| Immune checkpoint  inhibitor-related | 19 (2.1) | 5 (1.2) | 24 (1.8) |
| Surgery^*^ | 10 (1.1) | 8 (1.9) | 18 (1.3) |
| Pancreas divisum | 11 (1.2) | 4 (0.9) | 15 (1.1) |
| Diagnostic ERCP | 9 (1.0) | 5 (1.2) | 14 (1.0) |
| Pancreaticobiliary maljunction | 3 (0.3) | 7 (1.6) | 10 (0.7) |
| Chronic pancreatitis | 5 (0.5) | 1 (0.2) | 6 (0.5) |
| Duodenal papilla diseases | 1 (0.1) | 3 (0.7) | 4 (0.3) |
| COVID-19 | 3 (0.3) | 0 (0.0) | 3 (0.2) |
| Other endoscopic procedures^**^ | 2 (0.2) | 1 (0.2) | 3 (0.2) |
| Hereditary/Familial | 2 (0.2) | 0 (0.0) | 2 (0.1) |
| Abdominal injury | 2 (0.2) | 0 (0.0) | 2 (0.1) |
| Drug^***^ | 1 (0.1) | 0 (0.0) | 1 (0.1) |
| Autoimmune pancreatitis | 0 (0.0) | 0 (0.0) | 0 (0.0) |
| Others | 17 (1.9) | 9 (2.1) | 26 (1.9) |
| Total | 920 (100) | 428 (100) | 1,348 (100) |
| Etiology was not described in 7 males and 7 females.  *: includes cases due the stenosis of pancreatic anastomosis.  **: includes cases related to endoscopic ultrasonography-fine needle aspiration  ***: excludes cases with immune checkpoint inhibitor-related AP.  AP, acute pancreatitis; COVID-19, coronavirus disease 2019; ERCP, endoscopic retrograde cholangiopancreatography | | | |

| **Supplementary Table 5. Mortality rate according to the criteria of prognostic factors and CT grade** | | | | |
| --- | --- | --- | --- | --- |
|  |  | Prognostic factor criteria | |  |
|  |  | Negative | Positive | Total |
| CT grade criteria | Negative | 0.6% (18/3,013) | 13.2% (20/152) | 1.2% (38/3,165) |
|  | Positive | 2.4% (25/1,022) | 14.4% (27/188) | 4.3% (52/1,210) |
|  | Total | 1.1% (43/4,035) | 13.8% (47/340) | 2.1% (90/4,375) |
| CT, computed tomography | | | | |

| **Supplementary Table 6. Time to initiation of enteral nutrition** | | | |
| --- | --- | --- | --- |
| Time to initiation | Mild AP | Severe AP | All AP |
| Within 24 h, n (%) | 155 (18.9) | 155 (21.3) | 310 (20.1) |
| 25–48 h, n (%) | 199 (24.3) | 141 (19.4) | 340 (22.0) |
| 49–72 h, n (%) | 181 (22.1) | 117 (16.1) | 298 (19.3) |
| 4–5 days, n (%) | 146 (17.8) | 134 (18.4) | 280 (18.1) |
| 6–7 days, n (%) | 72 (8.8) | 56 (7.7) | 128 (8.3) |
| 8–14 days, n (%) | 48 (5.9) | 89 (12.2) | 137 (8.9) |
| After 14 days, n (%) | 18 (2.2) | 35 (4.8) | 53 (3.4) |
| Total, n (%) | 819 (100) | 727 (100) | 1,546 (100) |
| AP, acute pancreatitis | | | |

| **Supplementary Table 7. Type of antibiotics administered to AP patients** | | |
| --- | --- | --- |
| Type of antibiotics* | Mild AP | Severe AP |
| Cephem and β-lactamase inhibitor combination, n (%) | 721 (40.4) | 243 (21.8) |
| Carbapenem, n (%) | 345 (19.3) | 529 (47.5) |
| Cephem, n (%) | 450 (25.2) | 184 (16.5) |
| Penicillin and β-lactamase inhibitor combination, n (%) | 252 (14.1) | 146 (13.1) |
| Others, n (%) | 18 (1.0) | 12 (1.1) |
| Total, n (%) | 1,786 (100) | 1,114 (100) |
| *including overlapping cases. AP, acute pancreatitis | | |
